# Delegates of the Mississippi Valley Association

**Published:** 1859-07

**Authors:** G. W. Keely, Geo. Watt


### DELEGATES OF THE MISSISSIPPI VALLEY ASSOCIATION.

AT a called meeting of the Mississippi Valley Association, June 22d, 1859, the following members were appointed delegates to meet at Niagara, in accordance with the call published in the last number of the *Register:*

Drs. J. G. Hamill, Lancaster, O.; S. L. Hamlen, Cincinnati, O.; H. McCullum, Augusta, Ky.; A. G. Stipher, Decatur, Ill.; R. Peckover, Paris, Ky.; J. F. Mercer, Canada West; C. N. Woodward, Ripley, O.

On motion of Dr. Bonsall, it was

*Resolved,* That the delegates have power to fill vacancies in their own number, from any members of the Association who may be present.

On motion, adjourned. G. W. KEELY, Pres't.

GEO. WATT, Sec'y.

